# Self-Healing MXene- and Graphene-Based Composites: Properties and Applications

**DOI:** 10.1007/s40820-023-01074-w

**Published:** 2023-04-13

**Authors:** Atefeh Zarepour, Sepideh Ahmadi, Navid Rabiee, Ali Zarrabi, Siavash Iravani

**Affiliations:** 1 Department of Biomedical Engineering, Faculty of Engineering and Natural Sciences, Istinye University, 34396 Istanbul, Türkiye; 2 Department of Medical Biotechnology, School of Advanced Technologies in Medicine, Shahid Beheshti University of Medical Sciences, Tehran, 19857-17443 Iran; 3 Cellular and Molecular Biology Research Center, Shahid Beheshti University of Medical Sciences, Tehran, 19857-17443 Iran; 4 Centre for Molecular Medicine and Innovative Therapeutics, Murdoch University, Perth, WA 6150 Australia; 5 School of Engineering, Macquarie University, Sydney, NSW 2109 Australia; 6 Faculty of Pharmacy and Pharmaceutical Sciences, Isfahan University of Medical Sciences, Esfahān, 81746-73461 Iran

**Keywords:** MXenes, Graphene, Self-healing materials, Electromagnetic interference shielding, Wearable sensors

## Abstract

Today, self-healing graphene- and MXene-based composites have attracted researchers due to the increase in durability as well as the cost reduction in long-time applications. Different studies have focused on designing novel self-healing graphene- and MXene-based composites with enhanced sensitivity, stretchability, and flexibility as well as improved electrical conductivity, healing efficacy, mechanical properties, and energy conversion efficacy. These composites with self-healing properties can be employed in the field of wearable sensors, supercapacitors, anticorrosive coatings, electromagnetic interference shielding, electronic-skin, soft robotics, etc. However, it appears that more explorations are still needed to achieve composites with excellent arbitrary shape adaptability, suitable adhesiveness, ideal durability, high stretchability, immediate self-healing responsibility, and outstanding electromagnetic features. Besides, optimizing reaction/synthesis conditions and finding suitable strategies for functionalization/modification are crucial aspects that should be comprehensively investigated. MXenes and graphene exhibited superior electrochemical properties with abundant surface terminations and great surface area, which are important to evolve biomedical and sensing applications. However, flexibility and stretchability are important criteria that need to be improved for their future applications. Herein, the most recent advancements pertaining to the applications and properties of self-healing graphene- and MXene-based composites are deliberated, focusing on crucial challenges and future perspectives.

## Highlights

- Self-healing graphene- and MXene-based composites can be deployed in wearable sensors, supercapacitors, anticorrosive coatings, electromagnetic interference shielding, electronic-skin, and soft robotics.
- Self-healing graphene- and MXene-based composites have shown improved electrical conductivity, mechanical properties, healing efficacy, and energy conversion efficacy.
- Self-healing structures can open up considerable new horizons in the future of healthcare, sensors, electronics, robotics, supercapacitors/batteries, coatings, and biomedicine.

## Graphical abstract

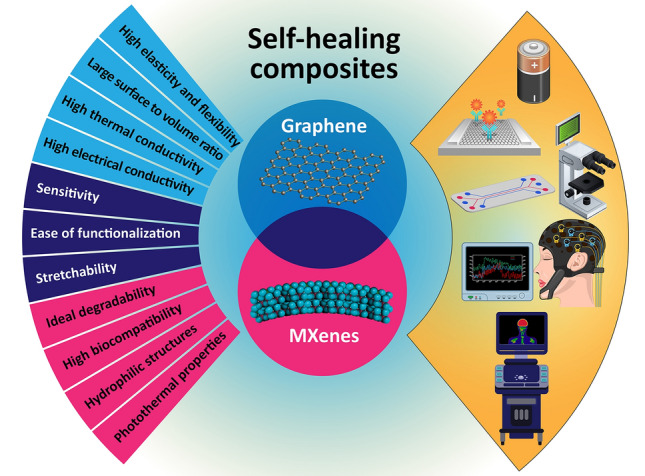

## Introduction

When damage occurred for a part of our body, a series of teamwork protective activities will start with the biological systems of the body to heal the injured part and regenerate its function. These events had been inspired a group of scientists the fabrication of self-healing materials that was introduced in 1970 by Malinskii et al. for the first time [1, 2]. During the healing process, the creation of a crack leads to the migration of healing agents toward the crack site due to the capillary effect followed by the catalytic crosslinking reactions that repair the crack [3]. The healing process is done via two different types of materials: (i) autonomic materials that have autoreactive properties for healing the crack and use the forces between different molecules or the cleavage and reconstruction of different chemical bonds for the healing process [1, 4], and (ii) non-autonomic materials that could act only in the presence of an external assistance like pH, light, or heat or an internal stimulation [5]. In this category, the reversible chemical bonds are usually created via two main types of dynamic bonds: supramolecular interactions (*e.g.,* hydrogen bond, metal–ligand complexation, π–π stacking, and ionic, hydrophobic, or host–guest interactions) and reversible covalent bonds [6].

So far different types of materials have been introduced for self-healing applications from different types of hydrogels to polymers, ceramics, metals, carbon nanocomposite, etc. Each of these materials has their specific features and limitations that could restrict their applications and make it important to apply some modifications on them. For example, autonomous healing in metal components is restricted and could be happened in high temperatures [7]. Polymers are the most extensively researched group of materials used for the fabrication of self-healing compounds with ideal processability, light weight, and chemical stability make them as a good choice to be applied in different fields from spacecraft, ships, and cars, to electronic and medicine [3, 8].

Application of nanomaterials in the field of self-healing opens a new era in this field [9, 10]. Indeed, nanomaterials could act as healing agents due to their migration ability to the crack area via an autonomous or stimulation reaction or could be applied with a healing component to improve other properties of the composite. They could facilitate the healing process via improving mechanical, biological, electrical, and functional properties, enhancing the half-life of materials, decreasing the cost, and finally elevating the comfort and efficiency [11, 12]. One of the interesting groups of these nanocomposites is two-dimensional (2D) nanostructures [13, 14], like graphene and MXene families [15–19].

**Fig. 1:**
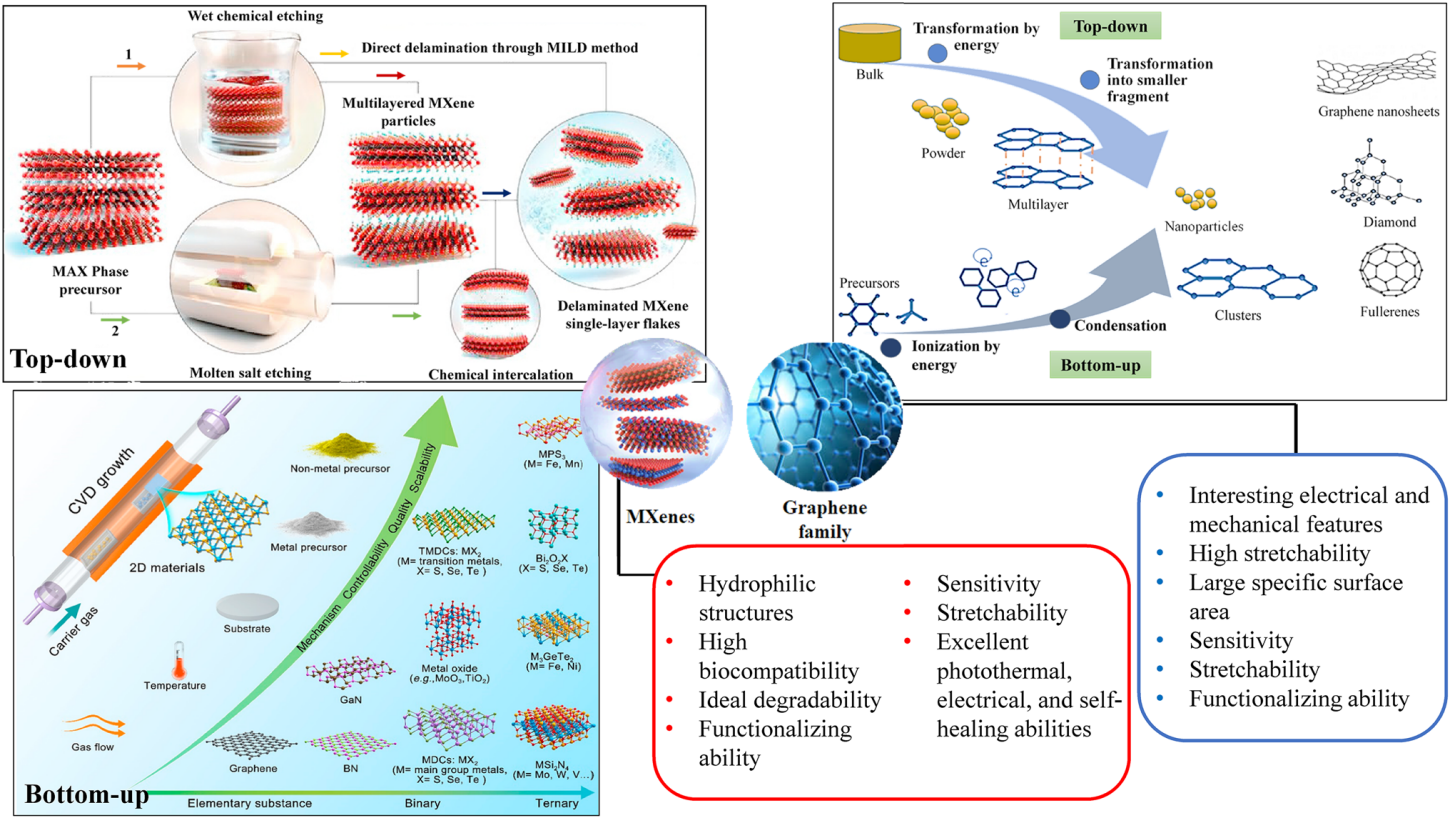
Schematic illustration of synthesis and advantages of MXenes and graphene family. Reproduced with permission from Refs. [49–51]. Copyright 2021 De Gruyter, 2020, American Chemical Society 2021, and American Association for the Advancement of Science

Graphene is fabricated from a single layer of carbon atoms in sp^2^ form with features such as large specific surface area, unique mechanical/electrical features, energy-absorbing capability, and thermal conductivity [20]. Based on the fabrication method and materials used, some other atoms are added to the carbon body of graphene that leads to the fabrication of other derivatives of graphene including graphene oxide (GO), reduced GO (rGO), carbon nanotube (CNT), etc. Due to their interesting features, the combination use of this family with self-healing structures could fabricate composites with interesting electrical and mechanical features. For instance, a porous conductive composite of rGO and a hydrogel-type shielding material was fabricated by Lai et al. [21] for the superb electromagnetic interference (EMI) shielding application. The fabricated composite had interesting features like excellent arbitrary shape adaptability, good adhesiveness, ideal durability, high stretchability, immediate self-healing responsibility, and superb electromagnetic feature (90.63 dB) with low reflection (6.41 dB) (Fig. 1).

MXenes are a new class of 2D materials that are composed of nitrides, carbides, and carbonitrides of transition metals with M_*n*+1_X_*n*_ (*n* = 1–3) formula, in which M is referred to the early transition metal (like Nb, Ta, Hf, Mo, V, Zr, Cr, Sc, Ti) and X refers to the carbon or nitrogen [22–24]. They were introduced in 2011 for the first time and have the capability of utilizing in different fields from energy evolution and environmental science to physics and biomedicine (especially nanomedicine) [25–30]. These are hydrophilic structures with several surface functional groups (such as oxygen, hydroxyl, and fluorine) and fascinating properties like high biocompatibility, good degradability, excellent aqueous dispersibility, and flexibly functionalized materials [31–35]. Utilizing the composites of MXenes with hydrogel could improve the sensitivity, stretchability, and flexibility of hydrogels from one side and prove the photothermal, electrical, and self-healing ability from the other side (Fig. 1) [36, 37]. The self-healing property of the MXenes has resulted from the recombination of the hydrogen bonds formed between different hydrophilic groups of MXene sheets and the interlayers’ water molecules, the same as what is happening for GO [38]. A variety of functionalized MXenes and MXene hybrids have been introduced with improved environmental stability, multifunctionality, stimuli-responsiveness behavior, biocompatibility/lower toxicity, contrast enhancement, and flexibility/stretchability for electronics, catalysis, energy storage, sensing/imaging, drug delivery, and cancer theranostics. Besides, MXenes and their derivatives can be further intercalated or hybridized with metals, graphene/its derivative, carbon dots, metal–organic frameworks (MOFs), and CNTs to improve their properties and functionalities [39–46]. Application of MXenes in the structure of a smart compression sensor led to an enhancement in the current, especially under heating conditions, and due to the contact created between different MXene nanosheets [47]. This self-healing feature along with the anticorrosion activity of MXene makes them suitable for using (in a composite form) as an anticorrosion coating [48].

Due to the fascinating properties and unique capabilities of self-healing graphene- and MXene-based composites, herein, the most recent advancements pertaining to the applications of these composites are deliberated, focusing on current challenges and future perspectives. In detail, we have described the current research performed on the application of self-healed graphene- and MXene-based composites in different fields from wearable sensors and supercapacitors to anticorrosive performance and EMI shielding materials, as well as the challenges related to the use of these materials, and their future probable applications.

## Self-healing MXene-Based Composites

### Wearable Sensors

**Fig. 2:**
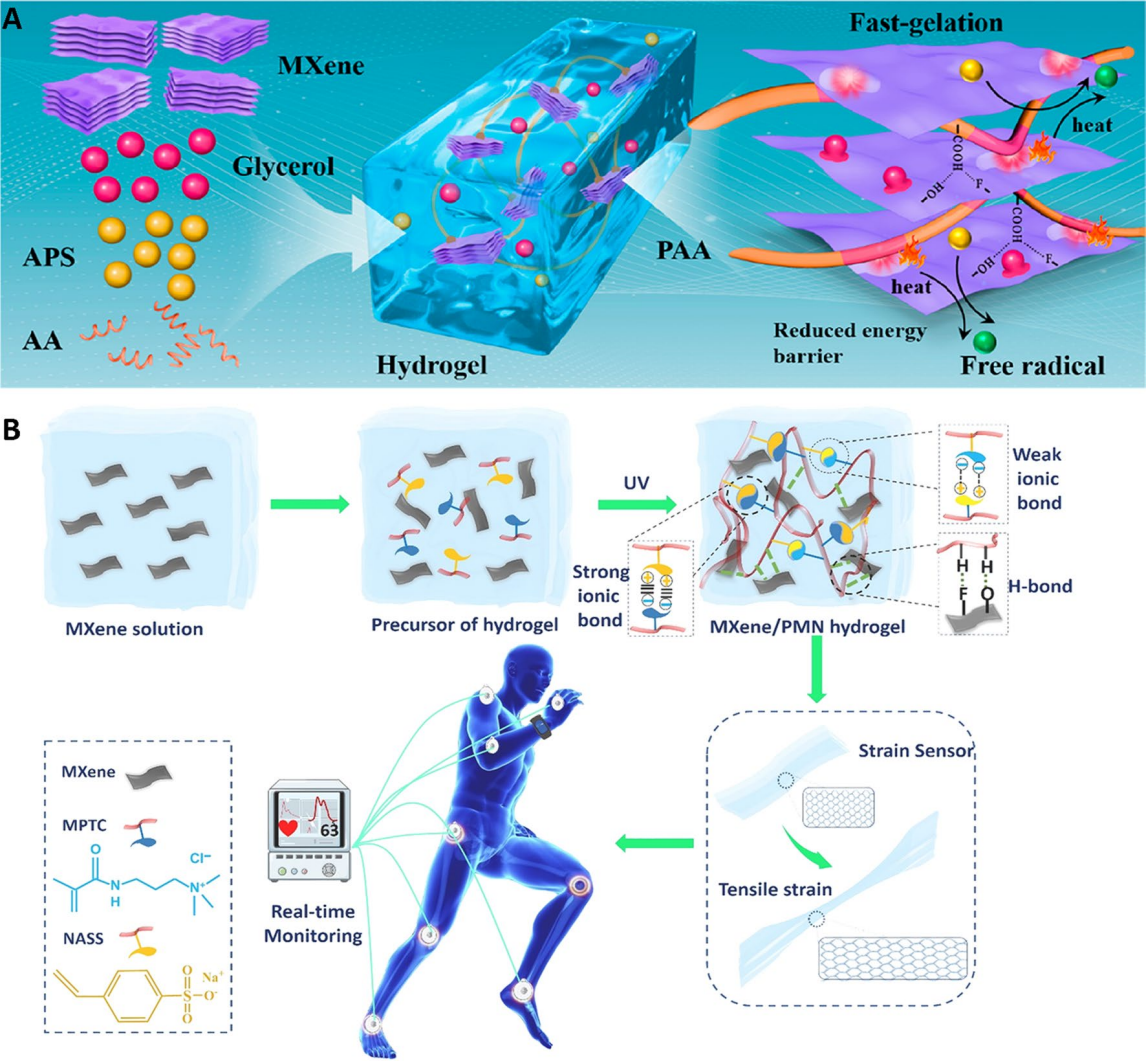
**A** The preparative process of PAA-MXene hydrogels, with related gelation mechanism. APS: ammonium persulfate; AA: acrylic acid. Reproduced from Ref. [36] with permission. Copyright, 2021 American Chemical Society. **B** The preparative process of MXene/polyampholytes (PMN) nanocomposite hydrogel using a one-step radical polymerization of 3-(methacryloylamino) propyl-trimethylammonium chloride (MPTC, cationic monomer) and sodium p-styrenesulfonate (NaSS, anionic monomer). Reproduced with permission from Ref. [52]. Copyright 2022 Elsevier

MXenes and their composites have been employed to design various novel hydrogel-based sensors with versatile applicability in personal healthcare monitoring, soft robotics, and electronic-skin (E-skin). In this context, one of the challenging issues is their self-healing capability as well as adhesiveness for full-scale monitoring of human motions [52]. In one study, MXenes have been used to activate the rapid gelation of various polymeric hydrogels initiating from monomers, namely N-isopropylacrylamide, poly(ethylene glycol) diacrylate, acrylamide, aniline, hydroxyethyl methacrylate, and N,N-dimethylacrylamide, acrylic acid [36]. They have been deployed for enhancing the stretchability of hydrogels, providing great opportunities to design novel MXene-based hydrogels with self-healing capacities for wearable and stretchable electronic applications. In an impressive study, Ti_3_C_2_T_*x*_ MXene with advantages of unique adhesion, mechanical features, and self-healing potential was employed as a 2D conductive crosslinker for initiating the polymerization and rapid gelation of polymeric hydrogels within several minutes (Fig. 2A) [36]. To fabricate polyacrylic acid (PAA)-MXene hydrogels, nanosheets of Ti_3_C_2_T_*x*_ were mixed with acrylic acid (the monomer) after addition of glycerol and ammonium persulfate. When MXenes with excellent photothermal features were incorporated in polymeric hydrogels encompassing unique phase-transforming properties, MXene hydrogel-based binary-layered actuator could be prepared [36]. However, more explorations are still needed for designing flexible sensors with self-healing, excellent sensitivity, and large stretchability. Despite the applicability of hydrogel-based wearable sensors in E-skin, healthcare monitoring, and human–machine interfaces, several crucial challenges still exist regarding their stability in a wide temperature range, adhesion, and sensitivity. Wang et al. [53] fabricated sensors utilizing oxidized sodium alginate, polyacrylamide, polydopamine-MXene (Ti_3_C_2_T_*x*_), and glycerol/water. These organohydrogels with unique mechanical features such as the tensile strength of 0.17 MPa and elongation at break of 1037%, exhibited self-healing performance (the self-healing efficiency was ~ 91%) along with significant sensing potentials (high sensitivity with gauge factor of 2.2) in a broad range of temperatures (− 20–60 °C). Polydopamine and viscous glycerin were introduced in these hydrogels to provide high adhesion properties. The high sensitivity could be obtained because of the combination of ionic and electron conduction, offering great opportunities for the detection of human movements at different temperatures and extreme conditions [53].

MXenes are disposed to oxidation owing to their abundant hydroxyls, resulting in instability in their conductivity particularly when they are employed in hydrogels. Qin et al. [54] introduced PAA/polyacrylamide/MXene/tannic acid hydrogels as strain sensors with improved conductivity, wherein tannic acid prevented MXenes from oxidation due to the excellent deal of pyrogallol groups. These biocompatible hydrogels displayed suitable tensile strength (0.251 ± 0.05 MPa), toughness (0.895 ± 0.16 MJ m^−3^), and elongation at break (560.82 ± 19.56%), showing good stretchability. The introduction of MXenes and tannic acid could provide good restorability and self-healing properties into hydrogel because of the presence of hydrogen bonds [54]. In addition, ultra-stretchable MXene/polyampholytes nanocomposite hydrogels were constructed with self-healing properties and multifunctionality (Fig. 2B) [52]. These hydrogels could be prepared through the one-step radical polymerization of cationic and anionic monomers. Remarkably, the simultaneous robust and weak ionic bonds-based crosslinking inside these hydrogels gifted them fascinating properties of stretchability, toughness, self-healing, and adhesiveness; MXenes were introduced in these composites to gain proper mechanical and conductivity features. The MXene/polyampholytes composites were employed to design wearable epidermal sensor with high accuracy and sensitivity for specific recognition of human motions. This sensor could be employed in designing detectors for real-time activity monitoring in patients, helping them for better management and treatment of their diseases. Developments in personalized healthcare monitoring, artificial intelligence, and human–machine interfaces can significantly help to design smart MXene-based sensors and next-generation devices with clinical and biomedical applications [52]. Similarly, epidermal sensor assembled from conductive MXene nanostructures were developed with good flexibility and conductivity along with self-healing, long-lasting moisture retention, and self-adhesiveness capabilities [55]. This sensor with durable stability could be applied for human motion biomonitoring. The nanocomposites were obtained from the conformal coating of the MXene nanosheet network using polymer networks of phenylboronic acid- and dopamine-grafted sodium alginate as well as polyacrylamide with a glycerol/water binary solvent as the dispersion medium [55].

MXene-based composites have been designed with flexibility, multifunctionality, and conductivity as wearable and stretchable strain sensors, showing excellent capabilities in E-skin, human detection sensors, and soft robotics [37]. But, important challenges still exist pertaining to the design of these composites to concurrently show enough stretchability, self-healing, sensing, and flexibility properties. Silicone polymer conductive composites were fabricated with good electrical conductivity utilizing MXenes (Ti_3_C_2_) and amino poly(dimethylsiloxane) [37]. Accordingly, the conductive composites with suitable tensile and self-healing potentials could be employed to design wearable strain sensors for specific detection of swallowing, pressing, speaking, etc. Notably, after repair, their tensile features and electrical conductivity were still remained ~ 98.4% and ~ 97.6%, respectively [37]. Wang et al. [56] developed a sensitive strain sensor using stretchable and self-healing conductive composites of *L*-citrulline-modified MXenes/polydimethylsiloxane, showing high sensitivity toward human activities/movements such as finger joint flexion, swallowing, and flexion. The addition of *L*-citrulline-modified MXenes could also enhance the electrical conductivity. The mechanical stress and strain of these composites could reach to ~ 4.78 MPa and 413%, respectively. In addition, after 24 h of repair without external stimulation, they exhibited suitable self-healing efficiency (~ 91%) owing to the dynamic disulfide and multiple hydrogen bonds [56]. Similarly, modified MXene-doped organosilicon conductive elastomer was constructed by crosslinking the hydrogen bonds and metal–ligand bonds; the reversible hydrogen bonds were generated between carboxyl groups in this composite [57]. After spontaneous repair, the polydimethylsiloxane@MXene recovered its original mechanical features (~ 91.7%). This composite with excellent stretchability and tensile strength can be deployed in soft robotics, motions monitoring, wearable strain sensors, etc. [57]. Zhang et al. [58] introduced a novel sensor with multiscale conductive layer structure through the spray of conductive materials to the elastomer with dynamic Diels–Alder bonds. This multiscale structure containing one-dimensional semi-embedded silver nanowires and 2D MXene nanosheets (the thickness of the MXene nanosheet was ∼1.8 nm) exhibited self-healing properties [58]. This sensor with a wide range of sensitivity (0.5–96%), suitable relaxation (~ 138 ± 5.8 ms), fast response (~ 71 ± 4.9 ms), and high durability could be deployed for the specific recognition of pressure (183–2260 kPa). In response to the pressure, this system was capable of real-time measuring signals, and the value of the examined stress could be encoded by the tensile instrument. This pressure sensor displayed high uniformity and stability at diverse range of stress with the same speed. Remarkably, the strain sensor displayed excellent sensing capabilities and stretching range (∼120%) compared to the single semi-embedded silver nanowire electrode. It can be deployed for detection of hand touching or human walking unique due to the unique mechanical and electrical features, opening a new window toward next-generation wearable electronics [58].

### Supercapacitors

MXenes with hydrophilicity, conductivity, tunable composition, and large specific surface area are attractive candidates in designing supercapacitors. Li et al. [59] developed self-healing micro-supercapacitors using size-dependent MXenes (Ti_3_C_2_T_*x*_) by spraying lateral size MXene nanosheets onto the cellulose paper. These MXene-based micro-supercapacitors with high flexibility could be assembled by sulfuric acid-polyvinyl alcohol (PVA) electrolyte. One of the important factors that could significantly affect the electrochemical function of these supercapacitors was the MXene nanosheet size. The capacitance of supercapacitors prepared by smaller lateral nanosheet could reach up to ~ 73.6 mF cm^−2^, which was higher than in the case of larger lateral nanosheets or combined smaller/larger nanosheets. Besides, MXene-based micro-supercapacitors with self-healing properties and excellent energy storage capacities were designed by applying polyurethane consisting of the large number of hydrogen bonds as the wrapped material; over 2000 charge/discharge cycles after 5th cutting/healing, the capacitance retention was still ~ 90% [59].

Recently, a wide variety of polymers have been employed as interlayers to enhance the flexibility of MXene films. In one study, Yu et al. [60] prepared PAA/chitosan (CS)/Ti_3_C_2_T_x_ hydrogel as flexible electrode materials. Self-healing and electrochemical properties of hydrogel can be tailored by regulating the content of Ti_3_C_2_T_x_ MXene. At the content of 0.3 wt% Ti_3_C_2_T_x_, the hydrogel electrode exhibited good capacitance (> 291.8 mF g^−1^), high stability, self-healing properties, and flexibility. Ti_3_C_2_T_*x*_ layered structure improved the transport of electrolyte ions. In addition, Ti_3_C_2_T_*x*_ increased the self-healing activity of the hydrogel, which could be redounded to the hydrogen bonds between Ti_3_C_2_T_x_ and the polymer. The results provide an appropriate way for the formation of electrically conductive hydrogels, which can be employed to design flexible supercapacitor electrodes and flexible electronic devices [60].

**Fig. 3:**
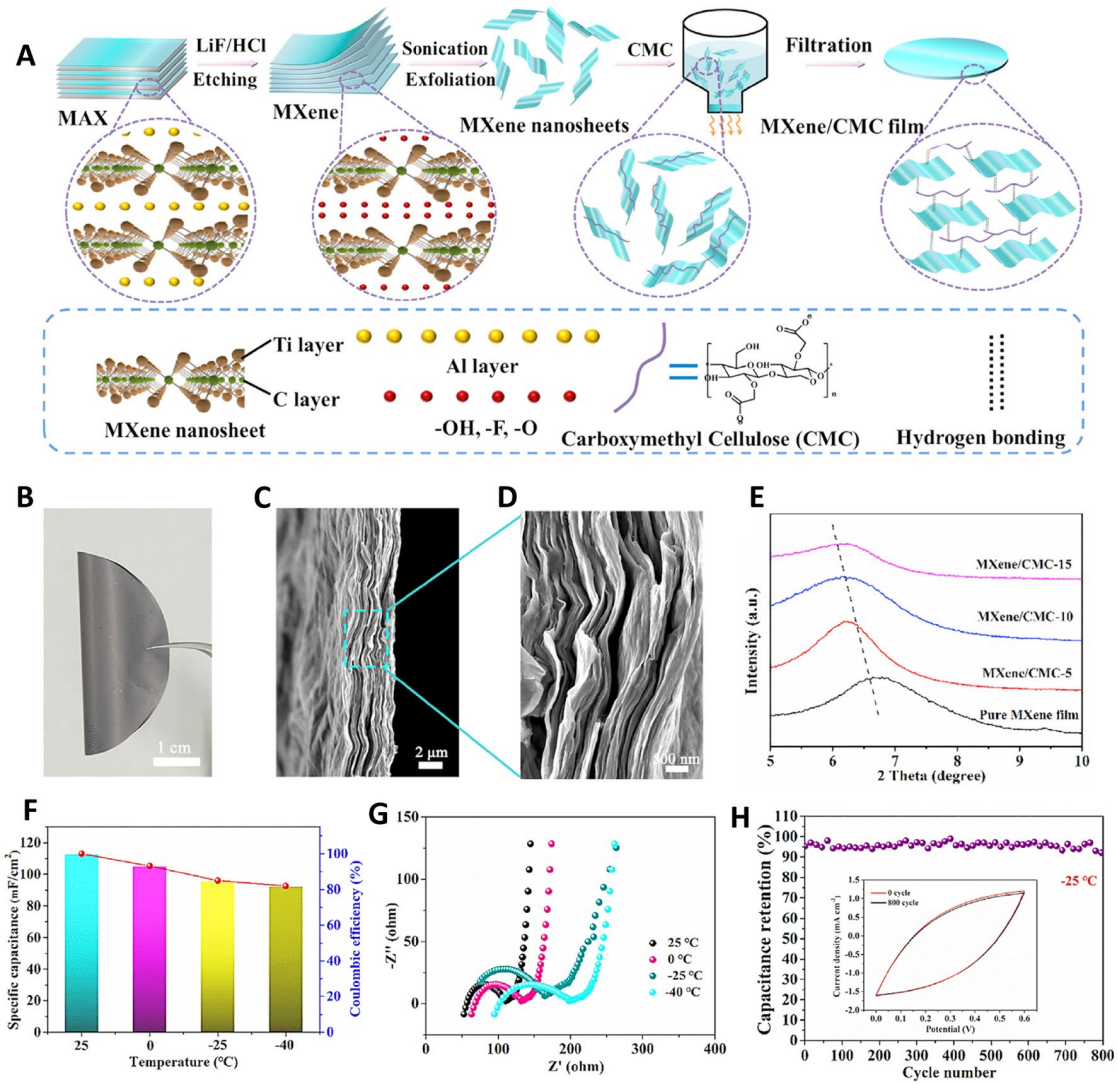
**A** The preparative process of MXene/CMC film. ** B** The image of the appearance of MXene/CMC-5 film. **C**, **D** Scanning electron microscopy (SEM) image of MXene/CMC-5 film. **E** X-ray diffraction (XRD) image of MXene/CMC films with different content of CMC. **F** Capacitance retaining of the supercapacitor under different temperatures. **G** Electrochemical impedance spectroscopy (EIS) spectra of the supercapacitor under different temperatures. H Cycle performance of the supercapacitor at 25 °C. Reproduced with permission from Ref. [61]. Copyright 2022 Elsevier

Researchers developed an assembly of flexible supercapacitors with mechanical deformation and a hydrogel of PVA/LiCl electrolyte. The presence of adequate LiCl could provide hydrogel with great mechanical softness and high conductivity; the use of LiCl can obstruct the formation of hydrogen bonds among PVA chains due to the binding of ^–^OH and Li^+^. Indeed, these properties cause the hydrogel to have low density and great stretchability. Besides, carboxymethyl (CMC) with high flexibility can be employed as interlayer spacers to efficiently inhibit the stacks of MXenes. The prepared films exhibited excellent mechanical flexibility and good conductivity to be applied as electrode materials of supercapacitors. The supercapacitor was formed from the cellulose film of the MXene/CMC electrode (Fig. 3). The supercapacitors combined highly conductive and flexible electrodes with hydrogel electrolytes that showed self-adhesion. The PVA/LiCl hydrogel displayed good self-healing capability; if the ends of the broken hydrogel were connected, the PVA/LiCl hydrogel could be healed. The supercapacitor had a great specific capacitance of 113.13 mF cm^−2^ during mechanical deformations [61].

### Anticorrosive Performance

**Fig. 4:**
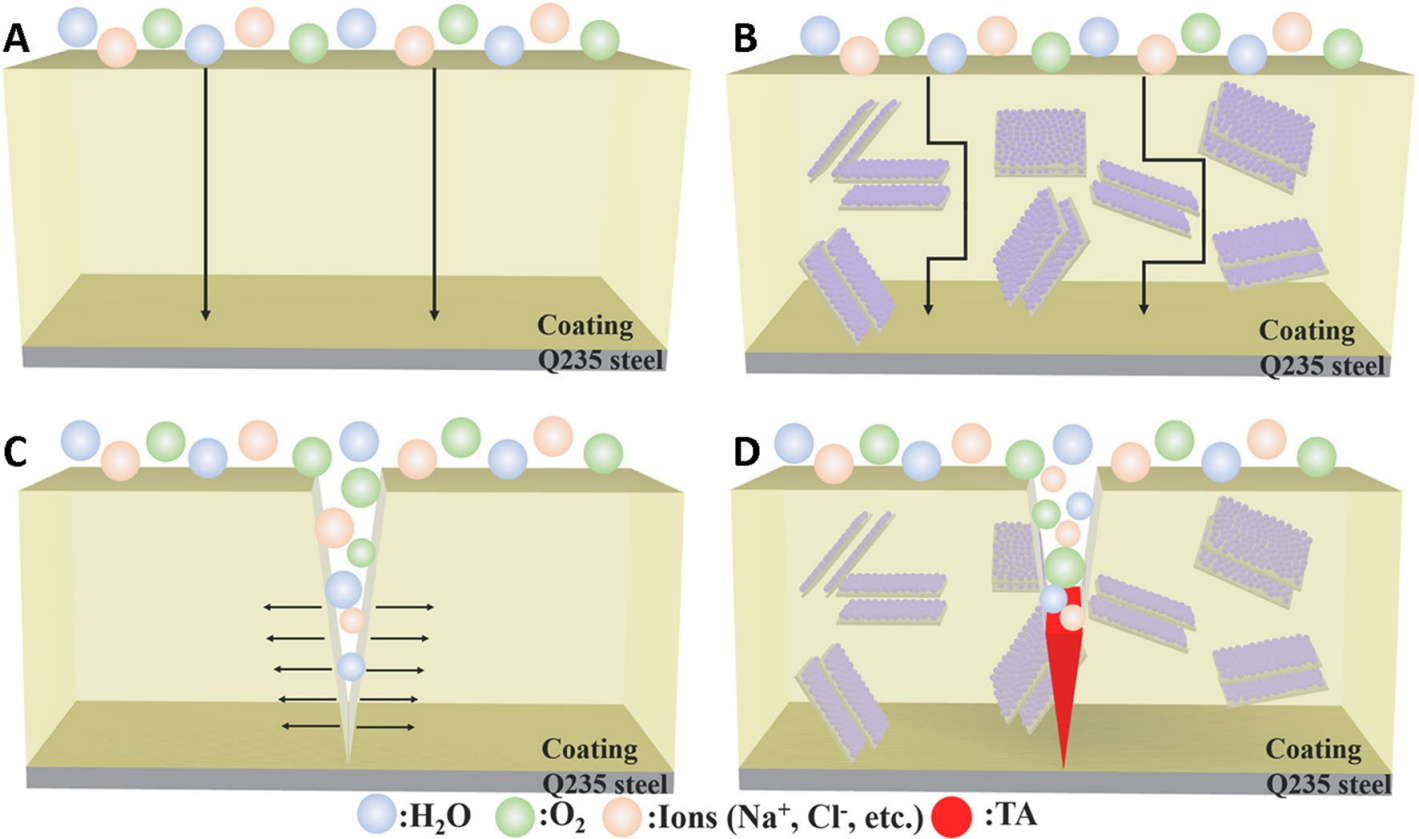
The anticorrosion and self-healing mechanism of tannic acid (TA)@MXene-SiO_2_-contained epoxy coating: the corrosion process without (**A)** and with **B** designed coating. **C** The process of crack propagation in the pure epoxy coating. **D** The epoxy/TA@MXene-SiO_2_ coating and its self-healing property; MXenes could successfully form a layer-by-layer matrix structure in this coating, providing excellent corrosion protection. Reproduced with permission from Ref. [62]. Copyright 2022 Elsevier

MXenes have been explored in construction of anticorrosive coatings; however, self-healing properties are one of the main challenges, which can guarantee the restoration of protective functions and the durability of these coatings in probable damages (especially in harsh corrosive circumstances). Sun et al. [62] constructed self-healing anticorrosive coatings with excellent anticorrosive property using MXenes and mesoporous silica (SiO_2_) nanomaterials loading tannic acid (an anticorrosion agent), protecting low-carbon steel from corrosion damages (Fig. 4) [62]. As a result, the impedance modulus was 2.826 × 10^6^ Ω cm^2^ at 0.01 Hz, and after 132 h of continuous immersion, it could reach the maximum peak value of 8.585 × 10^6^ Ω cm^2^. The electrochemical self-healing efficiency was 114.27 kΩ cm^2^ h^−1^, showing anticorrosive coatings with significant self-healing efficiency. After the damage scratch on the prepared coatings, the release of tannic acid encapsulated in mesoporous SiO_2_ nanomaterials was stimulated; this tannic acid could simply adsorb the steel in the crevice and react with Fe^2+^ to generate ferric tannates as a self-healing film [62].

### EMI Shielding Materials

**Fig. 5:**
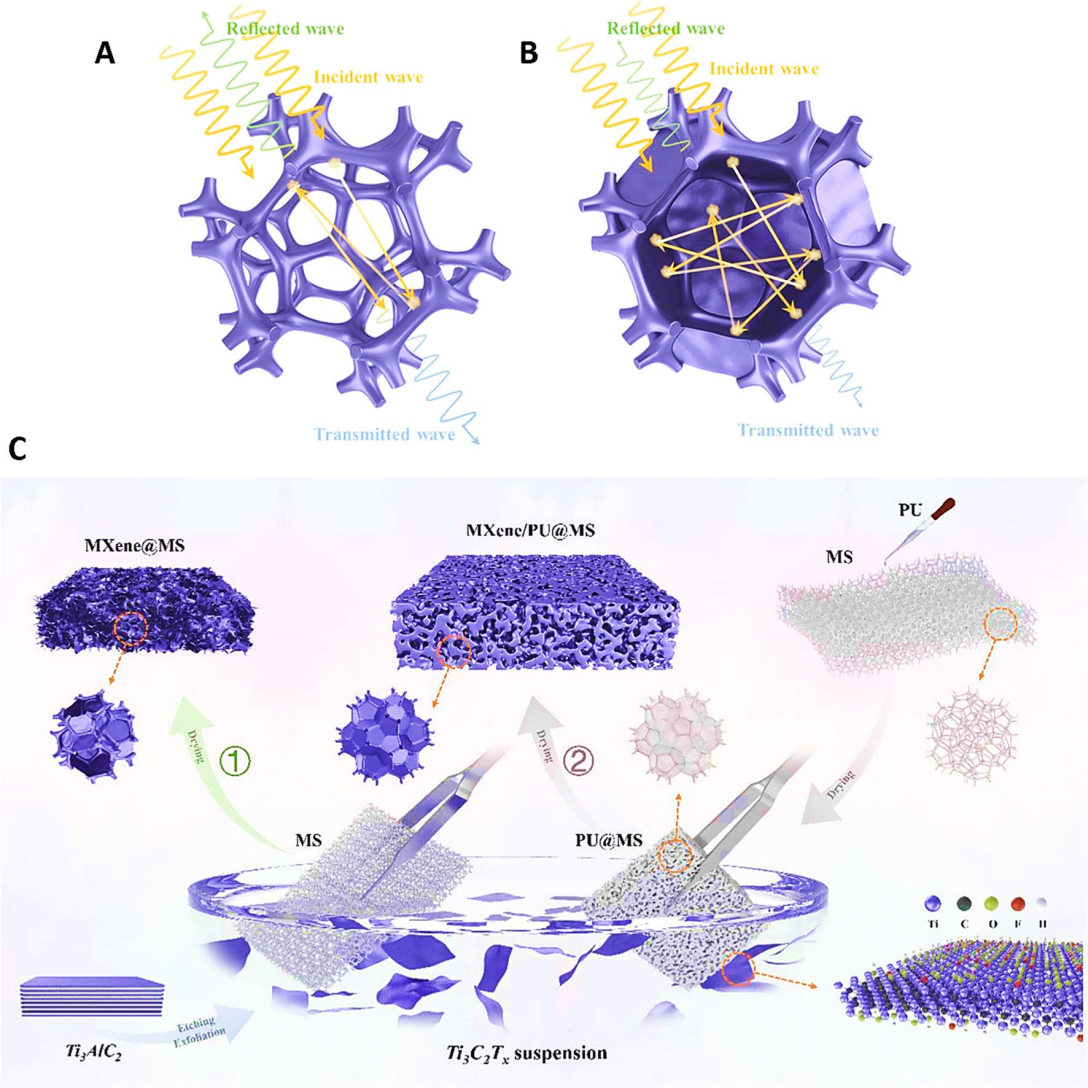
Three-dimensional (3D) porous composite sponges with skeletons wrapped by functional filler (**A)**, and pores covered by functional filler (**B)**, with related propagation route of electromagnetic waves. **C** The preparative process of (**1)** self-healing MXene-wrapped melamine sponge (MS) and (**2)** MXene/polyurethane (PU)@MS. Reproduced with permission from Ref. [71]. Copyright 2021 Elsevier

MXenes have been recently studied to design novel high-performance EMI shielding materials due to their unique electrical conductivity and layered structure; these materials can be considered as attractive candidates for flexible and wearable electronic devices [63–67]. However, the main challenge is designing multifunctional MXene-based composites with rapid healing properties, which can keep their mechanical and shielding functions [68]. For instance, powders of MXenes (Ti_3_C_2_T_*x*_) were introduced into paraffin matrix to obtain composites with EMI shielding effectiveness (SE) of ~ 39.1 dB at 60 wt% MXenes loading [69]. Additionally, MXene (Ti_3_C_2_T_*x*_)/epoxy composites were constructed using a solution casting technique, wherein the conductivity was up to 105 S m^−1^ and the EMI SE was up to 41 dB under 15 wt% MXenes loading [70]. In this context, several investigations have focused on EMI shielding MXene-based composites with good mechanical features and excellent electrical conductivity, along with appropriate EMI SE and self-healing properties. Lightweight, flexible, and self-healing EMI shielding structures were fabricated using MXene (Ti_3_C_2_T_x_) capsules in the porous sponge by applying a simple dip-coating technique (Fig. 5) [71]. Accordingly, the deployment of MXene membranes could form and cover the pores of the melamine sponges through the specific adjustment of the coating factors, which could further connect the porous skeletons into plenty of shielding capsules. In composite sponges, the enhancement of the internal area could improve the possibility of interactions between electromagnetic waves and the shield; thus, the generation of the incessant shielding walls thought the cover of the large voids among skeletons can be considered as promising tactic with high efficiency. The introduced sponges exhibited excellent EMI SE of 90.49 dB in the range of X-band (8.2–12.4 GHz). After the incorporation of polyurethane sandwich in these materials, the self-healing properties could be obtained; the healed sponges could still had significant EMI SE of 72.89 dB (the shielding efficiency was ~ 99.99%), which could support 1475 times the weight of the sample themselves. These MXene-based composite sponges with fascinating EMI shielding and self-healing features should be further explored for designing smart wearable devices [71].

**Fig. 6:**
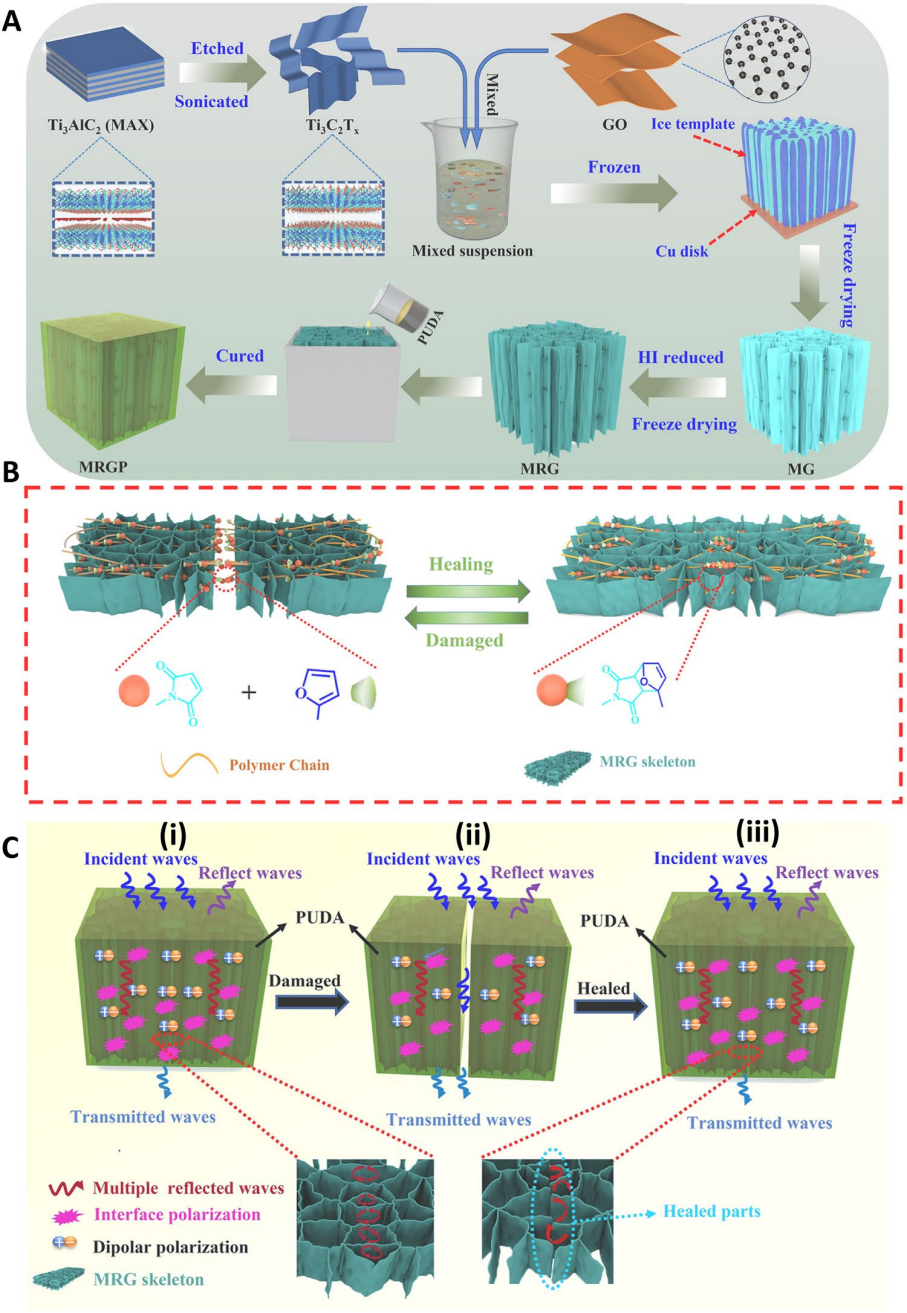
**A** Preparative process of MXene/rGO oxide/polyurethane (MRGP) composites. **B** Mechanism of heat-stimulated healing procedures in MRGP composites. **C** EMI shielding mechanisms of original (**i**) severed, (**ii)** healed, and (**iii)** MRGP composite skeleton. PUDA: polyurethane containing Diels–Alder bonds; MRG: MXene/rGO hybrid aerogel. Reproduced with permission from Ref. [72]. Copyright 2022 Elsevier

Several hybrid MXene-based composites were introduced for EMI shielding applications [72]. For instance, 3D conductive MXene (Ti_3_C_2_T_*x*_)/reduced GO composites were obtained through hydrothermal assembly and freeze-drying technique, wherein the epoxy resin was filled into the porous structure of MXenes by applying a vacuum-assisted impregnation tactic [73]. Besides, compressible polydimethylsiloxane-coated MXene foams were prepared for EMI shielding applications, with good flexibility (the EMI SE was ~ 53.9 dB) [74]. Despite the good flexibility of these composites, the capability of self-healing and long-term use is still a crucial challenge. On the other hand, when EMI shielding materials are damaged, their performance can be highly abridged, causing the possible electromagnetic threats/exposures [72, 75]. To overcome this problem, EMI shielding MXene (Ti_3_C_2_T_*x*_)/reduced GO hybrid aerogels with flexibility and self-healing properties were designed [72]. These aerogels with high electrical conductivity and 3D porous structures were fabricated via freeze-drying and chemical reduction pathways. After that, the dynamic crosslinked polyurethane containing Diels–Alder bonds was introduced into aerogels through a vacuum-assisted impregnation technique to obtain composites with significant EMI SE (~ 39.1 dB) at an ultra-low 0.46 vol% of MXenes and 0.65 vol% of rGO loading (Fig. 6). Diels–Alder bonds had crucial roles in self-healing properties of these composites. As a result, after 3 times of severing/healing cycles, the EMI SE could be recovered from 19.5 to 34.1 dB, with the healing efficiency of ~ 91.4%. These hybrid MXene/rGO composites can be employed in designing flexible and self-healing EMI shielding materials with long-term protection and electronic appliances [72].

## Self-healing Graphene-based Composites

Graphene is complemented through two forms, such as graphene attained from the chemical vapor deposition (CVD) method, as well as graphene derivatives comprising of GO [76, 77]. Graphene connections in the matrix can be through the mixture of graphene with matrix by molecular force, the connection of graphene and matrix through hydrogen bonds and polymer/graphene connection using reversible chemical bonds [78]. Different strategies have been introduced for synthesizing self-healing graphene-based composites, including simple mixing, in situ polymerization, Diels–Alder reactions, layer-by-layer self-assembly methods, and hydrothermal techniques [79]. Graphene-based materials have been developed with self-healing properties. They showed the healing of crashes produced in graphene using tensile load by molecular dynamics simulations. Typically, graphene-based composites can repair themselves when an external energy or stimuli is provided. In this context, some of the common healing conditions include heating, light radiation, microwave, and solvent-assistant self-healing. Additionally, simple contacting without any external stimuli and microcapsules is employed to heal the damaged composites [79]. The self-healing of cracks can occur in critical crack dislocation without any external force. According to the exclusive property of self-healing of single-layer graphene, they have predicted a future promise to sensor design, which causes it a great promise factor to future electronic devices [80]. However, bilayer graphene revealed high self-healing of cracks than a single layer. Self-healing can occur through a combination of the dangling bonds when inside the limit of the important crack initial dislodgment [81].

Overall, damage to polymer materials causes their breakage, thus reducing their stability. The self-healing of these materials is typically long and requires the help of various external stimuli [80, 82]. Graphene displays great strength and thermal conductivity, which can be applied to support polymers to make functional composites in different applications, such as electricity [83, 84]. Nevertheless, thermal conductive composites show more difficult healing due to the chemical alterations among the polymer and graphene fillers than the original polymers. However, rigid graphene fillers display a self-healing capability according to limited chain movement in the matrix based on polymer [85]. Besides, to increase the activity of the thermal conductive nanocomposites, excellent filler compound is combined, resulting in an accumulation of the fillers inside the matrix as well as reducing its limitations to rebuild the fillers for generating a network in the matrix after hurt. Thus, reducing the filler content and adjusting the formation of fillers are significant subjects in making self-healing thermal conductive composites.

**Fig. 7:**
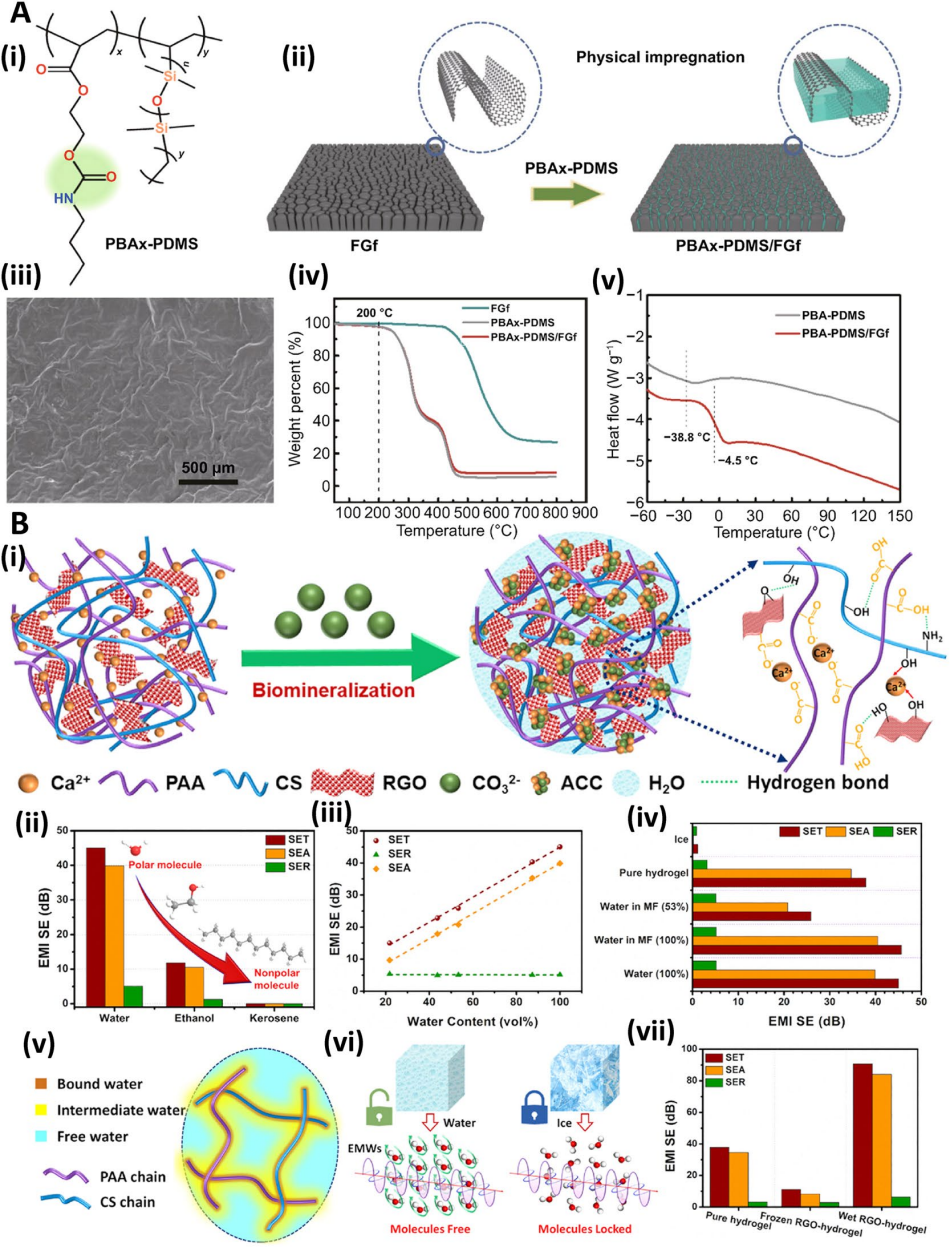
**(A)** Structure of PBAx-PDMS (**i**). Preparative process of PBAx-PDMS/FGf (**ii**), and the morphology of the introduced nanocomposite (**iii**). Thermogravimetric analysis plots of PBAx-PDMS and PBAx-PDMS/FGf (**iv)**. Differential scanning calorimetry curves of PBA-PDMS and PBA-PDMS/FGf **(v)**. Reproduced with permission from Ref. [86]. Copyright 2022 Springer Nature. **(B)** Synthesis of rGO composite hydrogel (i). EMI shielding efficiency (SE) variation with molecule polarity (ii). Difference of the EMI SE of H2O2 with the volume content in melamine foam (MF) (iii). Assessment of the EMI SE of water without MF, with MF, and the hydrogel (iv). The three different water types in the hydrogel (v), electromagnetic waves (EMW) transfer crossways water and ice (vi). The activity of the pure hydrogel along with the frozen and wet RGO-hydrogels (vii). PBA: 2-[[(butylamino)carbonyl]oxy]ethyl ester; PDMS: polydimethylsiloxane; FGf: folded graphene film; ACC: amorphous calcium carbonate; PAA: poly(acrylic acid); CS: chitosan. Reproduced with permission from Ref. [21]. Copyright 2021 Elsevier

Heat can improve polymer segment movement, a moderately mild path to self-repairing materials [86]. Researchers enabled GO-polyacrylic acid nanocomposite through crosslinking effects by ionic interactions, which were preserved at 45 °C to attain self-healing activity [87]. Although graphene filler was more stable than the polymer matrix, the deficiencies related to breaking inhibited self-healing (Fig. 7A). The effect of external stimuli was easy to produce swells on the damaged interface [86]. Low-strength electromagnetic waves pulsed at the earth’s natural frequencies have been revealed to enhance tissue healing and regeneration [88]. An electromagnetic wave is a type of energy that is established as healing; microwaves can be altered into heat. Coupling graphene with microwaves can make it suitable as an excellent energy absorber [84]. In one study, Huang and co-workers developed a graphene-thermoplastic polyurethane platform mended via microwave by heat, prompting the movement of a molecular segment. The combination of the microwave with graphene could increase the heat production; thus, Diels–Alder bond can be altered and tangled to attain a self-healing [89]. These electromagnetic waves are infrared light, sunlight, etc. GO could enhance the mechanical strength and increase tumor therapy. This material exhibited near-infrared (NIR) absorption, which changes light into heat.

Nanocomposites of polyurethane (HPU)-titanium dioxide (TiO_2_)/rGO were fabricated with dose-dependent mechanical features and high shape recovery ratio (91%-95%), along with the proper rate of shape recovery (1–3 min) under exposure to sunlight [90]. A great amount of rGO in the composite could aid in rapid and highly effective healing, while a great amount of TiO_2_ NPs (5–10 wt%) helped to attain virtuous self-cleaning properties. The nanocomposites exhibited intrinsic self-healing (~ 7.5–10 min) and suitable self-cleaning potential by eliminating methylene blue (~ 2–3 h). Sunlight is immeasurable energy and costs, and this nanocomposite with excellent potential can be deployed for versatile applications. This study is significant since sunlight is unlimited energy. However, infrared laser can be easier produced heat than the sunlight. A composite comprised of polyurethane based on Diels–Alder chemistry was connected with modified graphene nanosheets, displaying robust mechanical properties. This composite displayed ~ 96% healing efficacy in a short time [91].

### EMI Shielding Materials

Several studies have focused on semiconductors like rGO due to their exclusive electronic properties that are moderately helpful for EMI shielding. A combination of magnetic inclusions is essential to attaining effective absorption for EMI shielding [92, 93]. A “trigger-free” self-healable EMI shielding material containing rGO, MoS_2_, and Fe_3_O_4_/multi-walled carbon nanotubes (MWNTs) was constructed with ~ 99% reduction of electromagnetic waves by absorption [94]. These materials formed by MXene exhibited effective mechanical properties; however, the flexibility is still weak. The combination of 3D MXene-based structures with GO can improve the flexibility, wherein GO can be deployed as an efficient EMI material after reduction [95].

Hydrogel-type shielding composites were introduced using an rGO-constructed porous conductive network by applying a biomineralization-inspired technique. These hydrogel-type protective materials with porous conductive networks made of rGO exhibited excellent elasticity/permanency, outstanding desired adaptability/adhesion, and instant self-healing ability. Additionally, an excellent SE of 90 dB with a SE reflection of 6 dB could be instantaneously obtained utilizing rGO (4.7 wt%). The designed nanocomposites also displayed highly efficient healing activity that are carbon and MXene materials. The great effect of the conductive rGO network and the porous structure was crucial to such excellent shielding action. Polymer chains could alter the state of water, stimulate water molecules, and weaken electromagnetic waves. This self-healing platform offered a novel assay for forming good EMI shielding materials (Fig. 7B). The self-healing capability permitted rapid recovery from damage, and this type of graphene-based hydrogel displayed significant uses and flexibility to next-generation flexible electronics. Some of the 2D porous materials, such as MXene, have virtuous mechanical properties, although their flexibility of them is poor. The combination of MXene with the help of GO can overcome this problem. GO has a virtuous EMI shielding property after the reduction [21]. Sim et al. [96] fabricated graphene oxide/silver nanowire films and textiles with self-healing and flexibility properties for EMI shielding applications. Accordingly, significant EMI SE (~ 92 dB) could be obtained for the film with thickness of 18 μm; the precise EMI SE was 31 dB cm^3^ g^−1^ or 48,275 dB cm^2^ g^−1^ when normalized to film thickness. After damage, the mechanical strength and electrical conductivity of these films were reduced, and also their EMI SE was abridged from 72 to 56 dB at 8.2 GHz. However, their mechanical features were restored and EMI SE could be recovered to 71 dB after healing process. Besides, the textiles displayed EMI SE of ~ 30 dB, with significant flexibility and mechanical stability (no alteration in their performance was reported after 1,000 bending cycles) [96].

### Wearable Sensors

Graphene is a 2D material with exceptional electrical, thermal, and mechanical characteristics, along with good energy absorption properties [97, 98]. Thus, explorations have focused on evaluating self-healing graphene-based materials. Self-healing graphene-based materials with improved thermal/electrical conductivity, responsiveness to external forces, and the efficiency of healing exhibited suitable energy conversion efficacy [99]. The healing capability of the composites can be considered as one of the most properties of evolving wearable systems. For instance, self-healing and stretchable elastomer-graphene composites with remarkable mechanical and room-temperature healing properties were introduced due to the synergy of the microphase-separated structures with reversible hydrogen bonds [100]. These composites exhibited significant mechanical strength (~ 9.3 MPa), large extensibility (300%), healable efficiency (> 80%), and superb conductivity (~ 120 S cm^−1^), providing suitable platforms for manufacturing strain sensors with monitoring capabilities of tensile deformation and human motions. The prepared strain sensors maintained appropriate stability under ~ 20 cycles of stretching/releasing with 300% strain, which could be considered as promising candidates for next-generation wearable electronic devices [100].

Despite the advantages of graphene-based materials, their low stretchability may restrict their applications for wearable devices. Researchers developed a self-healing graphene hydrogel modified with polyurethane diol oligomer for wearable sensing systems. The polymer can act as a non-toxic plasticizer, which provided plentiful hydrogen bonds to create a solid state with high stretchability and quick healing capability. These sensors as resistive-type sensors using the modified hydrogels displayed great responsiveness to low-temperature changes (∆T ~ 0.2 °C) as well as the presence of ammonia (0.7–20 ppm) and nitrogen dioxide gases (0.8–3.5 ppm). In addition, these sensors could be stretched to 30%, and the healing capability of the hydrogels could recover the sensing ability of sensors (~ 90% in 30 s), offering the modified graphene-based hydrogels as promising materials for developing wearable sensors and E-skin [101].

One of the challenges is the existence of van der Waals interaction, which causes the aggregation of graphene nanomaterials in the aqueous environment and weakens the performance of composite hydrogels [102]. To overcome this challenge, 2,2,6,6-tetramethylpiperidine-1-oxyl (TEMPO)-oxidized cellulose nanofibers (TOCNFs)-graphene composites were employed in the hydrogel to prevent graphene from aggregation and increase the mechanical properties of the hydrogels. The electrostatic repulsion among TOCNFs and the hydrophobic interaction between graphene and TOCNFs caused the electron delocalization of graphene, leading to the attraction among the graphene and TOCNFs. This graphene-based hydrogel formed by the physical and chemical double crosslinking interaction can be deployed as a green wearable for monitoring human motion. The TOCNFs acted as a dispersant for graphene nanoparticles. The hydrogels exhibited suitable stretchability (~ 850%), electrical conductivity, and healing efficiency of 96% during 12 h. This hydrogel-based sensor demonstrated significant sensitivity, presenting excellent potential in the field of self-healing wearable electronic devices [102].

**Fig. 8:**
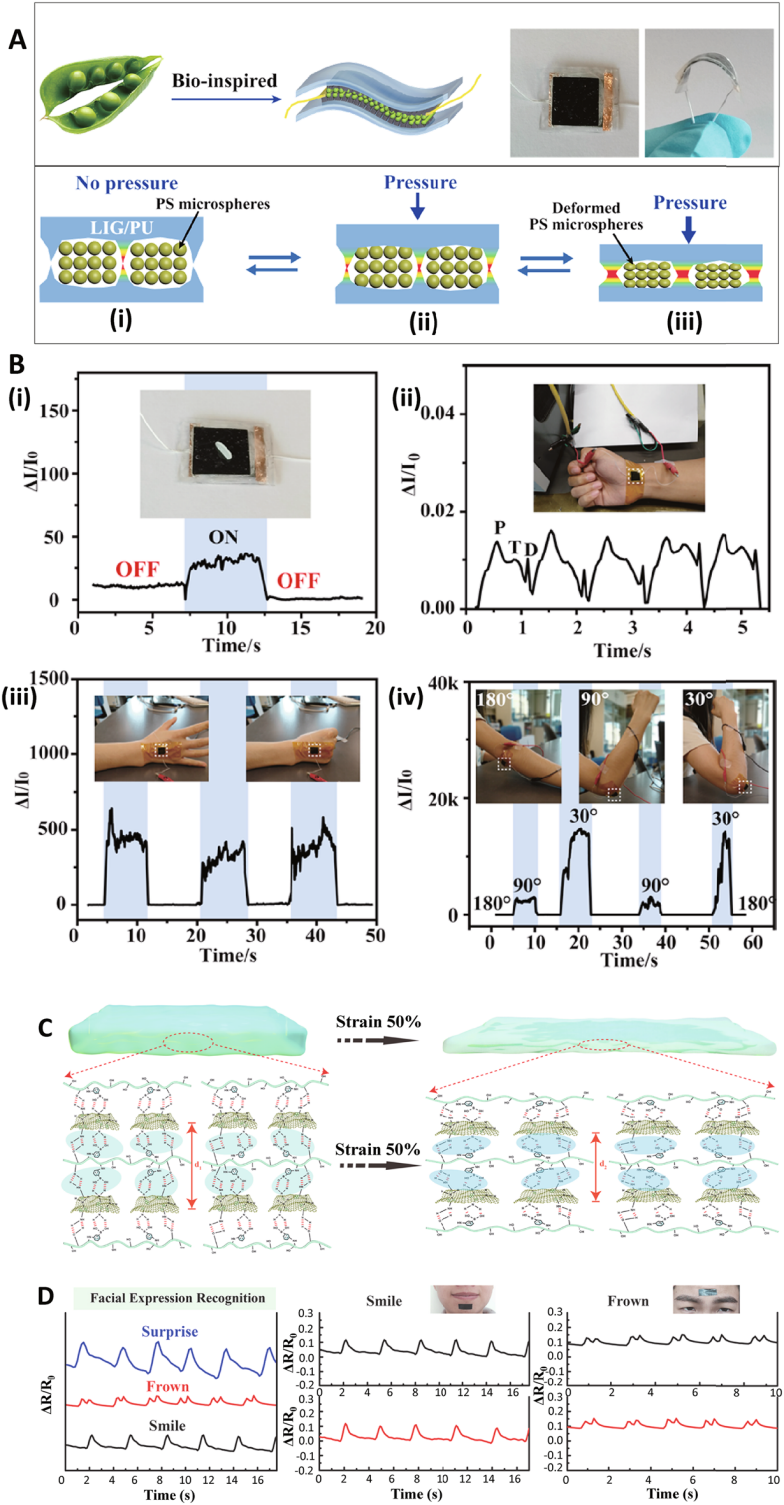
**A** Schematic illustration of the bean pod-inspired healable pressure sensor and its mechanism. **B** Wearable sensing applications of the pressure sensor in the detection of (**i**) rice as a light object, (**ii**) blood pulse on the wrist, (**iii**) hand clenching, and (**iv**) elbow bending. Reproduced with permission from Ref. [104]. Copyright, 2020 American Chemical Society. C Electrical properties of the GRSE-based sensors constructed from 1-pyrenamine-modified graphene layers. D Sensing activity of the sensor in human motion detection for recognition of smile and frown. Reproduced with permission from Ref. [105]. Copyright 2022 Elsevier

A wearable sensor was designed from PVA, GO, and polydopamine (PDA) nanocomposite for checking the large- and small-scale movements along with the physiological signals. GO exhibited stable distribution in different organic solvents due to the oxygen-containing groups, offering a good property for the synthesis of high-strength multifunctional nanocomposite hydrogels than graphene. This nanocomposite exhibited good electrical properties with a tensile stress of 146 Kpa and a conductivity of 5 mS cm^−1^. Besides, efficient self-healing could be obtained, with the electrical self-healing efficacy of ~ 98% and excellent self-adhesion onto surfaces of materials. The introduced hydrogel could be deployed to construct wearable sensors for specific detection of the signals, including large-scale motions in humans (e.g., stretching fingers joints) and small-scale motions (*e.g.,* breathing). In addition, the hydrogel was applied as self-healable electrodes to identify electrophysiological (ECG) signals. Thus, the GO-based sensor is anticipated to be applied for specific monitoring of human body motion [103]. In addition, a stretchy pressure sensor was designed with high stability and mechanical strength, which can be applied in wearable electronics [104]. Inspired by bean pod structure, this sensor was introduced with a micro-spacer core layer of polystyrene (PS) microspheres, sandwiched between two laser-stimulated graphene/polyurethane films (Fig. 8A, B). This self-healable sensor exhibited high stability and enhanced wide sensing range up to 100 kPa. After 3 cycles at room-temperature (RT) conditions, damaged systems were self-healed and capable to provide excellent sensitivity; the sensor can be deployed for checking human arterial pulse. Additional explorations ought to be focused on up-scalable production of these sensors with physiological diagnostic potentials [104].

Compared to the polymer materials, such as hydrogel and polyurethane, epoxy natural rubber (ENR) shows significant mechanical properties and good elastic deformation owing to the strain-induced crystallization (SIC), which can recompense for the restricted mechanical improvement of conductive (nano)composites. In one study, a self-healing graphene/rubber-based supramolecular elastomer (GRSE) was developed based on the effect of hydrogen bonds. Graphene nanosheets enhanced the conductivity and sensitivity of sensors. Accordingly, 1-pyrenamine (PA) was absorbed on the surface of the graphene nanosheet through the π^−^π conjugation to improve the amino groups and adhesion among the graphene sheet and elastomers. This wearable sensor provided high electrical conductivity (0.0029 S m^−1^), rapid (250 ms), and a low detection range (1%) property. Besides, the fabricated sensor could start its healing procedure at RT condition, with great mechanical strength and healing efficacy. The stress-sensing properties of this sensor can be employed for human motion detection (Fig. 8C) [105].

### Supercapacitors

A graphene-based supercapacitor was designed for storing energy, which could be recharged in short times. Graphene supercapacitor technology is safer than present battery technology since it can work without discharge or high temperature [106]. Graphene can be used as an effective candidate for designing wearable sensors and supercapacitors with multifunctionality [107]. Since the first graphene supercapacitor was introduced in 2008, major progress has been prepared in the development of novel graphene-based electrodes [108]. The significant electrical conductivity, unique mechanical features, and high thermal conductivity the graphene introduce this materials as an excellent gelator for manufacturing self-assembled graphene-based hydrogels with electromechanical performance [109]. The supercapacitor's activity of graphene was increased by doping them with oxygen, nitrogen, etc. It was observed that nitrogen doping into carbon increased the electrical conductivity [110].

**Fig. 9:**
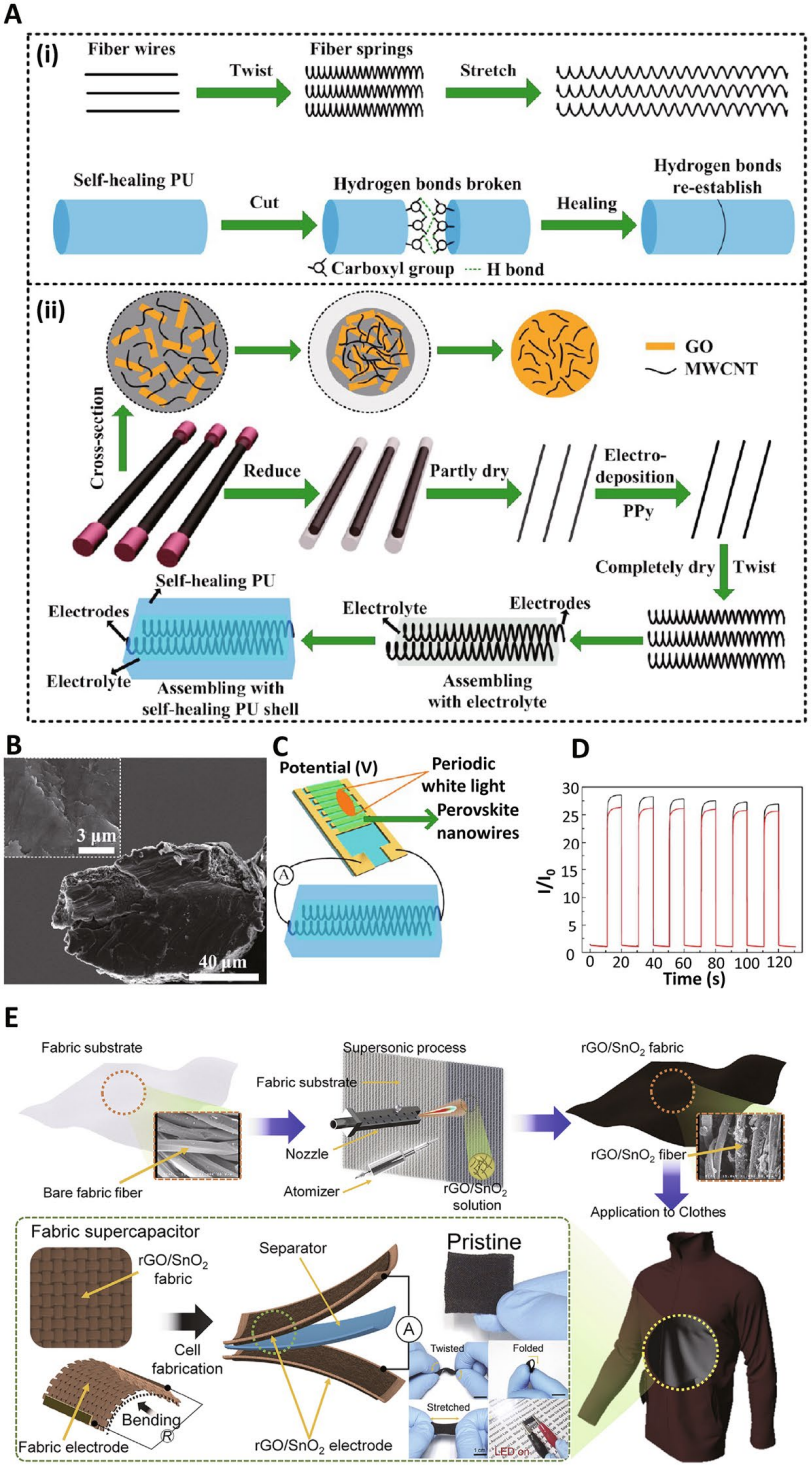
**A** Schematic illustration of the self-healable mechanism, along with the fabrication procedure of PPy/rGO/MWCNTs electrodes and self-healing supercapacitor.** (i**) rGO-based fiber wires can be twisted to springs. (**ii**) rGO/MWCNTs-based solution was added into pipes; then, rGO/MWCNTs composite fibers were formed. **B** SEM images of rGO fiber with the size of 100 μm and stacked morphology. **C** Schematic of the supercapacitor driving a detector of perovskite nanowires. **D** Photocurrent dependence on the time of the detector under illumination arises by the Reproduced with permission from Ref. [111]. Copyright 2017 American Chemical Society. (E) The preparative process of rGO/SnO2-containing droplets onto a fabric. Reproduced with permission from Ref. [112]. Copyright, 2021 Elsevierself-healing supercapacitor after a healing cycle.

Unfortunately, common graphene-based electrodes were thin; thus, it is impractical to re-connect the broken fibers joined precisely by visual assessment [111]. As a result, it is necessary to restore the electrochemical performance after the damage to the graphene-based fiber supercapacitor. rGO fiber-based springs can be employed as electrodes for self-healable supercapacitors. These fiber springs with a size of 295 µm are thick to rejoin the electrodes through visual assessment. Indeed, rGO-based wires containing polypyrrole (PPy)-decorated rGO/MWCNTs were warped to springs that can be stretched. This self-healable property comes from the hydrogen bonding of carboxylated PU **(**Fig. 9A, B). A self-healable supercapacitor could be developed by packaging fiber springs with a self-healing polymer outer shell, showing ~ 82% capacitance preservation after a great stretch of 100% after the 3rd healing. This study provided a novel approach for fabricating stretchable and self-healable [111].

Kim et al. [112] reported a new supercapacitor consisting of rGO and tin(IV) oxide (SnO_2_), in which rGO had efficient electrical conductivity and SnO_2_-informed energy storage abilities. On the other hand, supersonic spraying of rGO could decrease the peak ratio due to the self-healing effect. Supersonic spraying helped the good adhesion among the SnO_2_-decorated rGO flakes. The concentration effect of SnO_2_ on the electrochemical activity of the supercapacitors was also investigated; the best coating situations were recognized. Accordingly, the combination of rGO and SnO_2_ could increase the charge transport inside the electrode, finally enhancing the electrochemical performance. The porous construction of the fabric permitted the adequate electrolyte diffusion into the SnO_2_ or rGO to stimulate the function among the electrolyte with the electrode. The optimal sample displayed the greatest capacitance of 1008 mF cm^−2^, with the capacitance retention of ~ 93%. Stretching tests up to N¼ 1100 revealed the mechanical durability of the prepared supercapacitor, which was appropriate for generating energy storage systems on wearable fabrics (Fig. 9E) [112].

Self-restacking and accumulation of MXenes are commonly predictable throughout drying and electrode formation procedures due to the robust van der Waals interaction among neighboring nanosheets, which restricts the ion passage and decreases the active site in supercapacitors. Thus, the use of graphene between MXene layers can perform as a perfect spacer to inhibit the stacking among the MXene nanosheets, which aids to increase the electrochemical properties of the MXene [113–115]. A 3D composite containing MXene (Ti_3_C_2_T_x_)-rGO composite electrode was introduced, joining high specific surface area of rGO and excellent conductivity of the MXene to inhibit the self-restacking of the structure and resist the low oxidization of MXene. Besides, this composite exhibited good mechanical properties than pure rGO aerogels; the MXene/rGO/PU aerogel provided a great area-specific capacitance of 34.6 mF cm^−2^ at a scan rate of 1 mV s^−1^. The 3D composite also displayed an outstanding self-healing capability with specific capacitance retention of 81.7% after the fifth healing according to the PU properties. The formation of this self-healable can offer an assay for manufacturing long-life electronic devices needs [115].

### Cancer Therapeutics

**Fig. 10:**
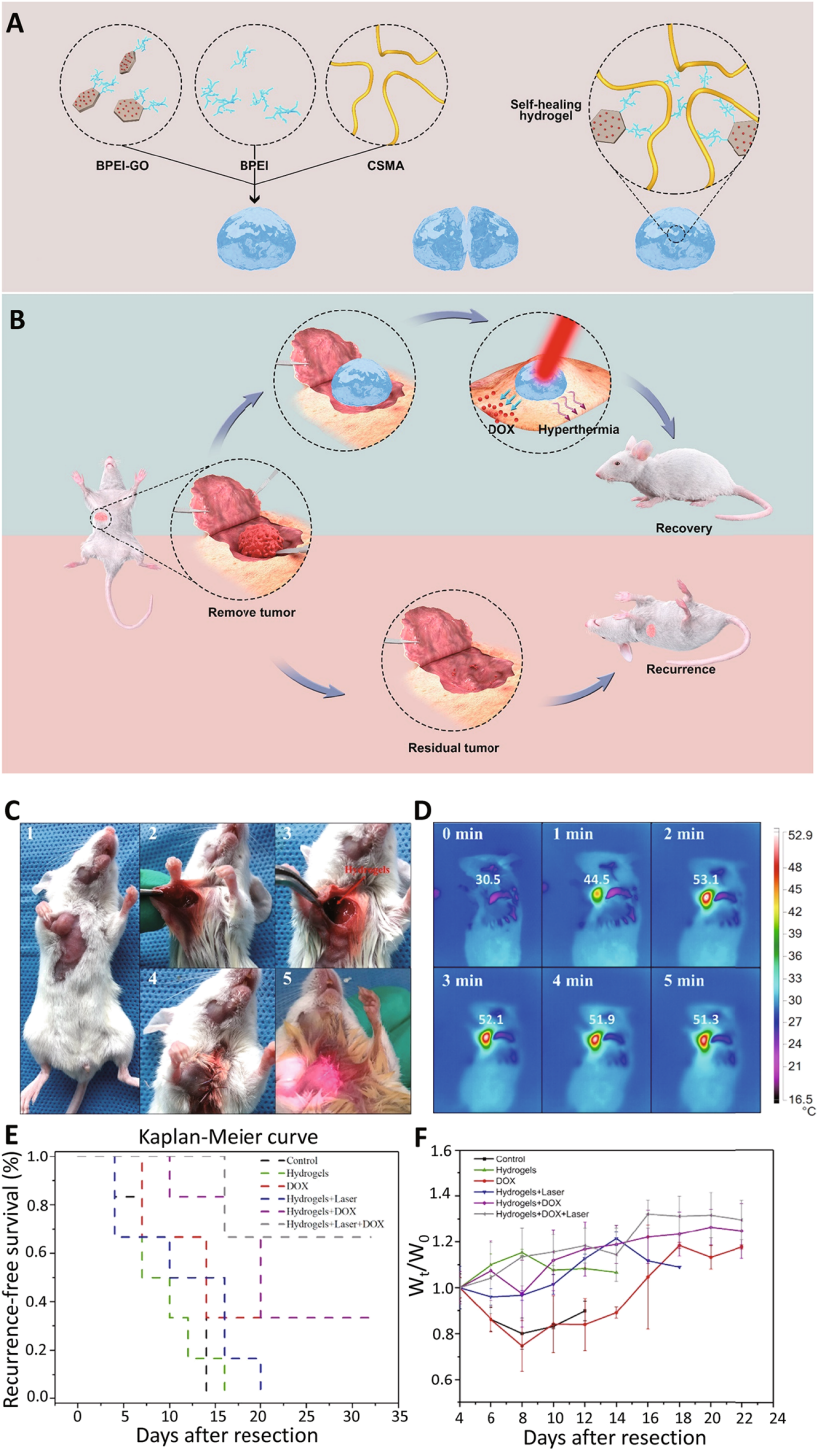
**A**, **B** Preparative process of CSMA/BPEI/BPEI hydrogels and their applications in targeted anticancer drug delivery and photothermal therapy of breast cancer. **C** Surgical procedure of tumor elimination and treatment: (1) the tumor volume was 200 mm^3^; (2) the tumor was removed; (3) hydrogel was entrenched; (4) incision was sewed; (5) mice were illuminated with near-infrared laser after 1 day. **D** Near-infrared imaging of mouse entrenched with hydrogels. **E** Kaplan–Meier survival curve plotting tumor recurrence. **F** Body weight difference throughout treatment. Reproduced with permission from Ref. [116]. Copyright 2019 American Chemical Society

Non-chemotherapeutic cancer and tumor therapy has gained significant consideration due to its lower side effects and unique targeting properties. GO nanoparticles may lose their stability (in vivo), because of possible aggregations when exposed in physiological environment after dispersion in solutions. Thus, surface modification techniques can be applied for improving the stability of these materials. Self-healing hydrogels were designed via Schiff-base linkage using chondroitin sulfate multialdehyde (CSMA), branched polyethylenimine (BPEI), and BPEI-conjugated GO (BPEI-GO) for targeted breast cancer therapy. Surface modification was deployed for enhancing the stability of these hydrogels using BPEI. These hydrogels could be doped in the network and provided targeted drug delivery and NIR-triggered photothermal therapy (Fig. 10). They displayed suitable self-healing (∼100%) and mechanical (7000 Pa) properties, providing improved cell killing efficiency (in vitro) with synergistic chemo-photothermal therapy. The combination of targeted chemotherapy (doxorubicin) with photothermal therapy using these hydrogels could reduce the recurrence of tumors to ~ 33.3%, compared to the examined doxorubicin-loaded hydrogels without near-infrared irradiation (~ 66.7%), local administration of free doxorubicin (~ 66.7%), and hydrogels with near-infrared irradiation (~ 100%) [116]. The results proposed the high efficiency of hydrogels in the recurrence inhibition of breast cancer, showing more efficient cancer therapy than in the metal-based nanomaterials [117].

A PEG-CMC/needle-like nano-hydroxyapatite (HAP)/GO nanocomposite hydrogel exhibited a construction with efficient injectability and good self-healing properties for tumor proliferation prevention and photothermal therapy. In vitro assessments revealed that GO was toxic to tumor cells, and HAP could prevent the proliferation of tumor cells; this composite was not transferred to the normal cells and did not led to cell damage. The breast cancer tumor-bearing mice were treated with a PEG-CMC/HAP/GO composite hydrogel (in vivo). This self-healing hydrogel successfully obstructed the tumor cell proliferation, showing suitable photothermal effects for targeted cancer therapy [118].

## Challenges and Future Perspectives

As mentioned above, both graphene and MXenes have interesting features that make them appropriate to be applied in the structure of self-healing composites; however, they also have some limitations that could restrict their clinical applications and necessitate conducting more research for overcoming these limitations. For instance, although evidence showed the short biocompatibility of MXenes (in cell culture tests), the long-term biosafety of these compounds is not completely assessed yet and more experiments are needed for confirming their safety for further biomedical uses. Indeed, due to the young age of this technology, our information about the immunogenicity, biocompatibility, biodistribution, and pharmacokinetics of different forms of MXenes is not comprehensive and several analyses on both small and large animal models are needed in this case [119, 120].

One of the important points for controlling different features of nanomaterials is their synthesis method. So far, a few attempts have been made for the fabrication of MXenes via bottom-up methods and they are commonly prepared via top-down methods leading to the preparation of multi-layer MXenes with less controllable structural and morphological features. Accordingly, still, the properties of naked MXenes with pure surface terminal groups have not been tested due to the absence of bottom-up methods for the fabrication of pure MXenes [120]. In the case of graphene, more information is available due to the existence of more research on this class of materials. Indeed, different improved bottom-up controllable methods have been introduced for the fabrication of different members of this family. The existence of abundant oxygen groups on GO nanosheets increases the mechanical strength of the sheets. Abundant hydrogen bonds were introduced at the interface between GO nanosheets with dynamic multiple hydrogen bonds and the polyurethane matrix to provide robust interfacial interactions. The adipic dihydrazide-modified GO sheets were combined into the polyurethane matrix, and the hydrogen bonds were presented at the interface among the polyurethane matrix. This nanocomposite exhibited excellent mechanical strength (~ 78 MPa), with rapid self-healing potential (~ 88% for 24 h), and the polyurethane/GO network could be employed to design flexible smart robots as well as flexible devices with multifunctionality [121]. This nanocomposite with rapid self-healing potential could recover up to ~ 50% of its elasticity in 1 min without any curing agents [122]. However, there are still some limitations in this issue as well; for example, evidence about the biocompatibility of graphene-based materials is diverse, in some cases they are biocompatible and, in others, they are toxic. Besides, their application in self-healing materials confronts some limitations; the most important of them is non-repeatable self-healing features. Indeed, more research is needed to design and fabricate effective composite materials with ideal self-healing features. Moreover, the mechanism of action and the molecular structure of these materials should be completely studied for better optimization of different properties, and for enhancing the applicability and practicability of the fabricated materials. Besides, this research could be beneficial for the introduction and fabrication of multifunctional compounds especially those that will be used in biomedical fields. The other important challenge of composite of these two materials with other materials is providing the maintenance between their mechanical properties and self-healing ability [2]. Remarkably, most of the present works are in the theoretical stages that have far distance to the real situations. Indeed, long way is imagined to pass for mimicking even a simple biological healing process and we are still in the first steps of self-healing materials production [1]. Alternatively, the ideal way of healing is one without the interference of external factors and stimuli and is completely independent. As a result, there is a need for special design and synthesis of nanostructures. Systems that depend on external sources, including heat, light, etc., have several challenges. In a study developed by researchers, a modified graphene/polyurethane composite was developed with self-healing property through infrared laser. However, it required a high temperature (150 °C) from a laser source to enable Diels–Alder chemistry [122]. In addition, researchers demonstrated that polyacrylamide hydrogels based on GO could be self-healed at RT condition. Still, their healing efficacy decreased considerably without the presence of moisture conditions, from 92 to 45% [123].

One of the main challenges in designing hydrogels used in electroactive tissues is the deficiency in electrical conductivity and adhesiveness [123–126]. Jing et al. [124] introduced composite hydrogels constructed from chitosan (as a biocompatible and biodegradable polymer) and graphene oxide with self-adhesive and healing features along with suitable electrical conductivity (reached to 1.22 mS cm^−1^); these composites were designed by incorporation of mussel-inspired protein polydopamine. The significant stability and mechanical properties as well as adhesiveness (the adhesive strength was increased by 300%), self-healing, and fast recovery capabilities could be achieved due to presence of hydrogen bonding, covalent bonds, π−π stacking, and supramolecular interactions in these composites. Because of the enhancement in human embryonic stem cell-derived fibroblasts and cardiomyocytes cell viability and proliferation, after the utilization of these composite hydrogels, they can be considered as promising tissue engineering materials with both self-healing and electrical conductivity properties [124].

Graphene-based nanocomposite materials were developed with self-healing potentials without external forces, which had quick electrical recovery from damages. They had steady electrical resolution even when the cyclic test was done up to 150 cycles. These materials can be employed to design bio-electronic sensor devices with excellent permanency [127]. In one study, graphene-based composites were fabricated to design electromyogram sensor showing autonomous self-healing properties throughout the mechanical deformation of ~ 50% through the recovery of electrical paths in polymer networks; the procedure did not require any force. Besides, these composites exhibited stable electrical activity in extending tests after healing in the absence of bilayer structure [127]. However, the combination of healing and functional abilities in robust and light-weight materials was an important limitation. Combining a polymer in a graphene ultralight network can form highly electrically conductive material with self-healing properties. The application of these composites could sense pressure after damage without an external source. After damage, the crack could be healed in 1 day without source. Furthermore, the network's self-healing was assumed by creating dynamic bonds among the oxygen and boron on a silicon substrate. The graphene network also performed as an electrical route offering electrical conductivity. This crosslinking does not need any external source, and these materials can be employed as promising candidates for biomedical and robotic applications [128].

Despite these limitations, the self-healing composites of graphene and MXenes are promising materials with bright future, especially for the fabrication of innovative and smart products in biomedical applications. They could be applied for the fabrication of E-skin, anticorrosion coating, and intelligent sensors. For instance, a type of self-healing composite of bio-based polyschiff vitrimers and GO was synthesized by Jia et al. [129] to be applied as a temperature and fire warning sensors that could be used for the critical fire risk and related perilous circumstances. In another case, self-healing composite of GO and polyurethane was fabricated to be applied as stimuli-responsive shape memory materials that could be applied in different parts of the body [130]. Remarkably, a variety of fascinating properties can be introduced in MXenes and their composites by suitable functionalization/modification strategies. For instance, Haddadi et al. [131] reported the construction of amino-functionalized MXene (Ti_3_C_2_) nanosheets through an etching technique and modification process utilizing 3-aminopropyltriethoxysilane. As the corrosion inhibitors, cerium (Ce^3+^) cations were introduced and encapsulated within MXene nanosheets to obtain self-healing epoxy composite coatings, showing good corrosion protection performance [131]. In addition, self-healing vinyl-functionalized GO-based nanocomposite hydrogels were introduced with improved mechanical properties; GO was functionalized with vinyl groups utilizing (3-mercaptopropyl) trimethoxysilane through a silanization technique. This self-healing potential of these hydrogels could be due to the chemical crosslinks and physical interactions in the polymer network [132].

In recent years, E-skin with interesting features like self-healing, versatile sensory capability, and stretchability is one of the interesting fields of science that will have prospective applications in soft robotics, artificial intelligence devices, and personalized medicine. E-skin has similar functionalities as human skin such as self-healing properties, stretchability, and flexible sensory ability, which can be deployed in soft robotic systems, artificial intelligence devices, etc. [133, 134]. These are fabricated based on utilizing strain sensors working based on converting the mechanical stimulations to detectable signals. A composite of MXene (Ti_3_C_2_Tx)/PVA hydrogel electrode with specific features like self-healing capability and stretchability was fabricated for E-skin applications. The presence of MXene in the structure of this E-skin leads to an enhancement in its self-healing ability and conductivity. This electrode shows excellent stretchability and self-healing properties (healing time ≈ 0.15 s). The sensor could recover its activity after a self-healing test, showing excellent potential for human motion monitoring [135]. A composite of ionic liquids (ILs) and MXene-binary polymer network was also fabricated in another study that has interesting capabilities like excellent adhesion, strong tolerance against harsh environments, ideal mechanical properties, high sensitivity to both pressure and strain, and light-responsive self-healing ability that appropriate it for multifunctional flexible sensors [136]. These along with several other samples show that application of both graphene-based materials and MXenes in the structure of composites is a promising strategy for inducing both healing and mechanical features and making them ideal for versatile applications in the near future. However, we need to conduct a deep comprehensive study to clearly find out the different features of these two classes of materials, their long effects on the environment and also the effects of other components on them, and their effects on our body and genome for their biomedical applications.

Although MXenes have distinct features and have shown promising potential for various applications, there are still some obstacles in the way of their commercialization and clinical implementation [137]. One of the most crucial challenges in using MXenes for versatile applications is their limited stability due to their high reactivity causing possible degradation, restricting their applicability. However, the stability of MXenes can be improved by suitable functionalization or hybridization with other materials such as polymers, carbon-based materials, and metal/metal oxide [138, 139]. In addition, the large-scale fabrication of MXenes can be challenging; more elaborative studies are still warranted to find sustainable and environmentally friendly methods for the synthesis and processing of MXenes [140]. Notably, long-term toxicity and systematic clinical assessments should be prioritized for research, especially for commercialization and future clinical applicability [141].

## Conclusions

Graphene and MXene-based composites have been developed with fascinating self-healing properties. Different literature has been accompanied on the limitations of graphene and MXenes on strength, mechanical potential, and response to external forces in the self-healing field. In addition to rheological recovery tests, the gelation, injectable, stretchable, and cut-heal properties ought to be comprehensively evaluated; the pH-responsive behavior and swelling behavior/swelling kinetics of the self-healing hydrogels should be analytically studied. Graphene-based materials have exceptional electrical, mechanical, and thermal properties and good energy absorption. Although more information is needed in graphene applications, especially regarding the applicability of self-healing graphene-based composites and understanding of their related mechanisms, the design of high-performance structures using polymers in combination with graphene and its derivatives still needs additional explorations.

It is necessary to fully understand the related mechanisms of self-healing materials based on graphene and MXenes to design novel composites with multifunctionality. On the other hand, MXenes are exceptional, because they have great interlayer spacing, good electrical conductivity, unique architectures, and virtuous thermal stability. According to the great conductivity of MXenes, their composites displayed good conductivity, which could be applied in the self-healing process. MXenes exhibited greater electrochemical properties than other 2D nanomaterials such as graphene. Some of their properties such as abundant surface terminations and great surface area are important to evolve biomedical and sensing applications. However, flexibility and stretchability are crucial aspects that should be improved for their future practical applications. Several conductive graphene- and MXene-based composites with suitable elasticity and stretchability can be developed only after specific optimization processes as well as suitable surface modifications; these composites can be employed in different fields of sensitive strain sensors, health monitoring, E-skin, soft robotics, etc. Tough, the fabrication of these conductive materials with outstanding mechanical features, self-healing features, and sensitivity/selectivity is still an important challenging issue. Additionally, when the concentration of MXenes in a polymer matrix increases, the conductive network of MXene fragments is not break in the anticipated strain range. However, some of their limitations can be overwhelmed through the surface functionalization/modification as well as hybridization of MXenes; surface modification is essential to increase the attraction among the MXenes with the polymeric matrix. Due to their high reactivity, it is possible to perform several surface modification reactions by hydroxyl groups on the surface of MXenes.
